# Vibrational spectra of neutral and doped oligothiophenes and polythiophene†

**DOI:** 10.1039/d2ra07625j

**Published:** 2023-02-13

**Authors:** Stewart F. Parker, Jessica E. Trevelyan, Hamish Cavaye

**Affiliations:** a ISIS Neutron and Muon Source, STFC Rutherford Appleton Laboratory Chilton Didcot OX11 0QX UK stewart.parker@stfc.ac.uk

## Abstract

We have measured the infrared, Raman and inelastic neutron scattering (INS) spectra of a series of oligothiophenes (bithiophene, terthiophene, quarterthiophene, sexithiophene and octithiophene) and polythiophene, both pristine and after doping with iodine. The spectra of the pristine (*i.e*. neutral) systems show a rapid convergence towards the spectrum of polythiophene, such that the spectra of sexithiophene and octithiophene are almost indistinguishable from that of polythiophene. The spectra, in combination with periodic density functional theory calculations, have also provided the first complete assignment of polythiophene. In contrast to the infrared and Raman spectra that show dramatic changes on doping, the INS spectra show only small changes. Isolated molecule DFT calculations show that the molecular structures are not greatly modified on doping and since the INS spectrum largely depends on the structure, this does not change much. In contrast, as shown by others, the electronic structure is greatly modified and this accounts for the major changes in the infrared and Raman spectra.

## Abstract 2

The vibrational (infrared, Raman, inelastic neutron scattering) spectra of the oligothiophenes show a rapid convergence towards the spectrum of polythiophene. DFT calculations show that the molecular structures are not greatly modified on doping.

## Graphical abstract

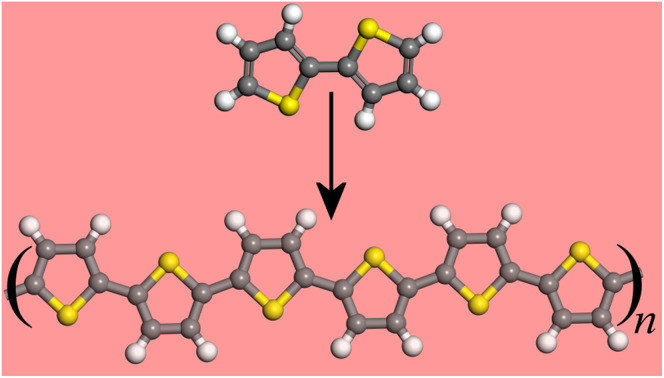

## 1. Introduction

Since the discovery^1^ of the first conducting polymer (Br doped polyacetylene), the field has rapidly expanded.^2^ The interest in conducting polymers arises from their attractive properties. These include favourable electronic properties and component versatility, and potentially, low production and installation costs because they are lightweight and solution processable.^3^ This makes large area and flexible devices possible and enables them to be used in a range of electronic applications, including solar cells,^4^ organic light-emitting diodes (OLEDs)^5^ and sensors.^6^ There are a wide range of materials available, however, the oligothiophenes^7^ and polythiophene^8^ have attracted intense interest due to their chemical stability^6^ and electrical conductivity, in particular when doped with iodine.^9^

The oligothiophenes have long been used as model compounds to understand the spectroscopy of polythiophene, both in the pristine^10–18^ and doped state.^10,19^ There are also many studies of doped polythiophene itself *e.g*.^10,19–24^ The spectral assignments have been supported by computational studies at levels of theory ranging from empirical force field^25^ to semi-empirical^13^ to Hartree–Fock^15^ to density functional theory (DFT).^17,24,26–31^

Most of the spectroscopic studies have used infrared and Raman spectroscopy. In these, the intensity of a mode is determined by the electro-optical response of the material (dipole moment derivative and polarisability derivative respectively). These properties are drastically altered on doping, which makes determining structural information from the spectra very difficult. In contrast, the intensity of a mode in inelastic neutron scattering (INS) spectroscopy is determined by the amplitude of motion of the atoms in a mode and the incoherent scattering cross section.^32^ Neither of these depend on the electronic structure: the former depends on the molecular structure and the latter is a fundamental property of the scattering atom(s). This means that INS spectroscopy is sensitive to the local structure in the material. There have been several studies of the oligothiophenes that included INS data^15,17,26,27^ but none have directly made the link to polythiophene. The INS spectrum of polythiophene has only been reported twice,^33,34^ one of which was low resolution and of limited energy transfer range.^33^

In this paper we show how the INS spectra of the oligothiophenes evolve into that of polythiophene and then use DFT calculations of the unit cell of polythiophene to provide a complete assignment of the material. We then investigate how doping changes the spectra and compare DFT calculated INS spectra of doped materials with the experimental spectra to determine both the degree of doping and gain insight into the structural changes caused by doping.

## 2. Experimental

**Fig. 1:**
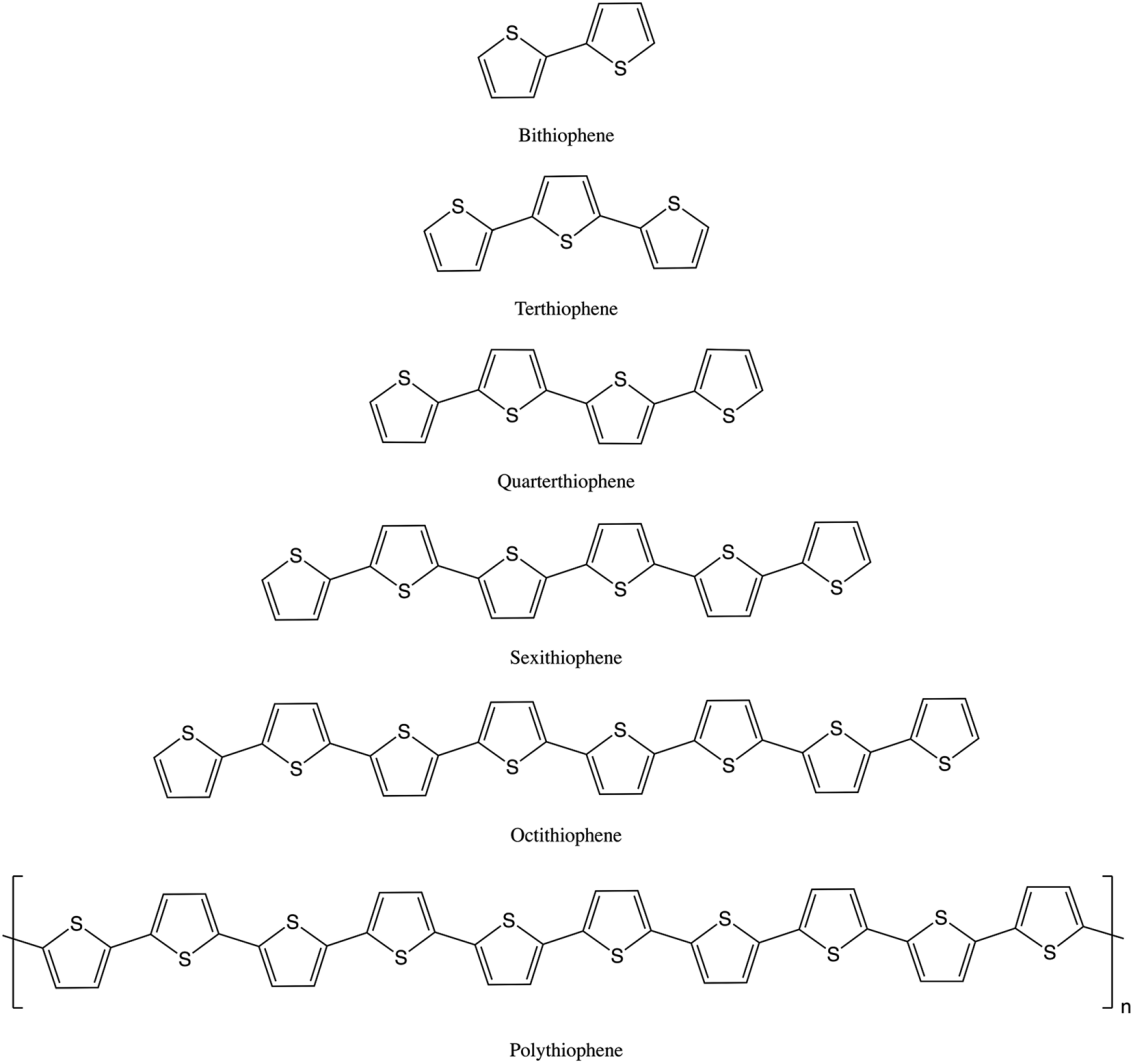
Structures of the oligothiophenes and polythiophene.

2,2′-Bithiophene (2T), 2,2′:5′,2′′-terthiophene (3T), 2,2′:5′:2′′:5′′,2′′′-quarterthiophene (4T) and 2,2′:5′:2′′:5′′,2′′′:5′′′,2′′′′:5′′′′,2′′′′′-sexithiophene (α-sexithiophene, 6T) were purchased from Aldrich, 2,2′:5′:2′′:5′′,2′′′:5′′′,2′′′′:5′′′′,2′′′′′:5′′′′′,2′′′′′′:5′′′′′′,2′′′′′′′-octithiophene (α-octithiophene, 8T) was purchased from Tokyo Chemical Industry and polythiophene (PT) was purchased from Rieke Metals. The characterisation of the PT sample is presented in the ESI.† All were used as received. The structures are shown in Fig. 1. Iodine doping was attempted by using a KI-I_2_ aqueous solution but was unsuccessful except for 6T. The materials were doped by exposure to I_2_ vapour in a desiccator. Iodine content was determined from the weight gain. The final loadings of iodine-per-thiophene ring were: 3T 1.19, 4T 1.38, 6T 0.20, PT 0.46. We were unable to dope 2T by either method.

Infrared spectra (4 cm^−1^ resolution, 256 scans, 8 times zerofilling) were recorded at room temperature with a Bruker Vertex 70 Fourier transform infrared spectrometer using a Bruker Diamond single reflection total internal reflection (ATR) accessory. The spectra were corrected for the wavelength dependent pathlength using the Bruker software. Raman spectra were recorded with a Bruker FT-Raman spectrometer (64 scans at 4 cm^−1^ resolution with 10–200 mW laser power at 1064 nm, 8 times zerofilling). INS spectra were recorded at <10 K using the TOSCA^35,36^ spectrometer at ISIS. TOSCA provides excellent quality spectra in the 0–1800 cm^−1^ region. At higher energy transfer, the spectra suffer from both resolution broadening (the resolution degrades with increasing energy transfer^32^) and are dominated by combination modes of the fundamentals and the lattice modes. This is particularly the case in the C–H stretch region, such that the spectra do not offer any useful information in this region. This is explained in more detail elsewhere.^37^

Isolated molecule DFT calculations were carried out with Gaussian 09.^38^ The 6-311g(d) basis set with the B3LYP functional was used. Infrared and Raman spectra were generated from the Gaussian output using GaussSum.^39^ Dispersion corrected (DFT-D) periodic calculations were carried out using the plane wave pseudopotential method as implemented in the CASTEP code.^40,41^ Exchange and correlation were approximated using the PBE^42^ functional with the Tkatchenko–Scheffler (TS) dispersion correction scheme^43^ within the generalized gradient approximation (GGA). The plane-wave cut-off energy was 1000 eV. Brillouin zone sampling of electronic states was performed on 10 × 10 × 4 Monkhorst–Pack grid (30 *k*-points). The equilibrium structure, an essential prerequisite for lattice dynamics calculations was obtained by BFGS geometry optimization after which the residual forces were converged to ±0.00755 eV Å^−1^. Phonon frequencies were obtained by diagonalization of dynamical matrices computed using density-functional perturbation theory^44^ and also to compute the dielectric response and the Born effective charges, and from these the mode oscillator strength tensor and infrared absorptivity were calculated. In addition to the calculation of transition energies and intensities at zero wavevector, phonon dispersion was also calculated along high symmetry directions throughout the Brillouin zone. For this purpose, dynamical matrices were computed on a regular grid of wavevectors throughout the Brillouin zone and Fourier interpolation was used to extend the computed grid to the desired fine set of points along the high-symmetry paths.^45^ The atomic displacements in each mode that are part of the Gaussian and CASTEP outputs, enable visualization of the modes to aid assignments and are also all that is required to generate the INS spectrum using the program AbINS.^46^ It is emphasised that for all the calculated spectra shown the transition energies have not been scaled.

## 3. Results and discussion

### 3.1 Assignment of the spectrum of polythiophene

The structures of the oligothiophenes have all been determined by single crystal X-ray diffraction (2T,^47,48^ 3T,^49,50^ 4T,^51^ 6T,^52^ 8T ^53^) and that of polythiophene itself by X-ray powder diffraction.^54^ All of the oligothiophenes are planar with either *C*_2v_ (odd-T) or *C*_2h_ (even-T) symmetry. PT itself is also planar.^54^ All of the oligothiophenes and polythiophene adopt the alternating structure (“S-up, S-down, S-up…”) shown in Fig. 1.

**Fig. 2:**
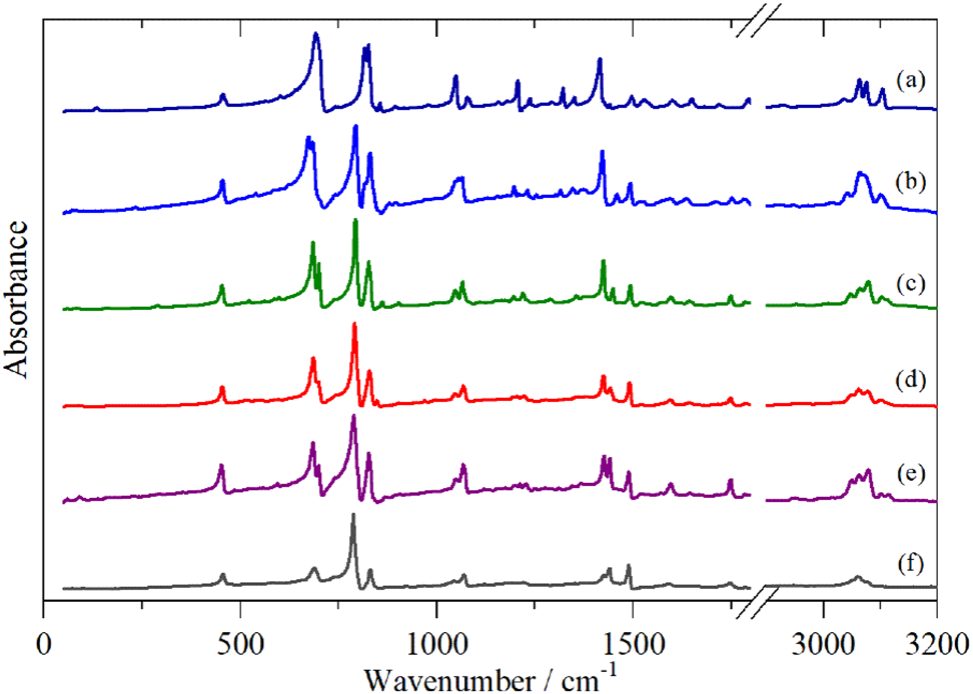
Infrared spectra of the oligothiophenes and polythiophene. (a) 2T, (b) 3T, (c) 4T, (d) 6T, (e) 8T and (f) PT.

**Fig. 3:**
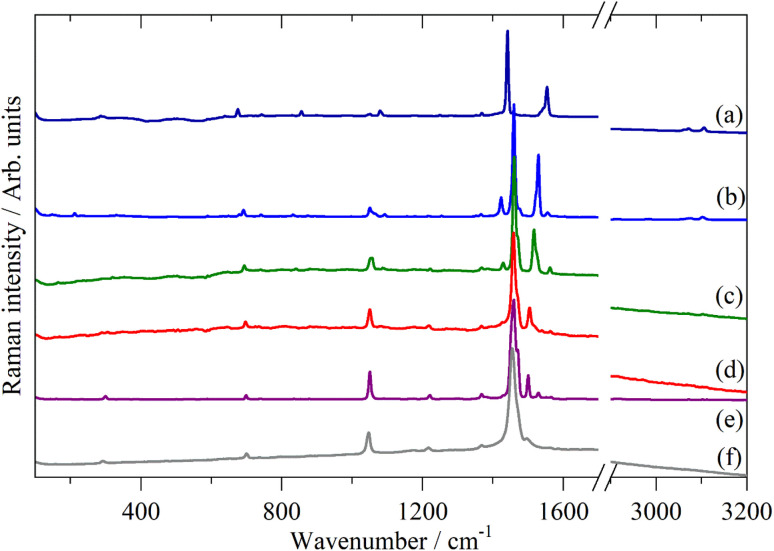
FT-Raman spectra of the oligothiophenes and polythiophene. (a) 2T, (b) 3T, (c) 4T, (d) 6T, (e) 8T and (f) PT.

**Fig. 4:**
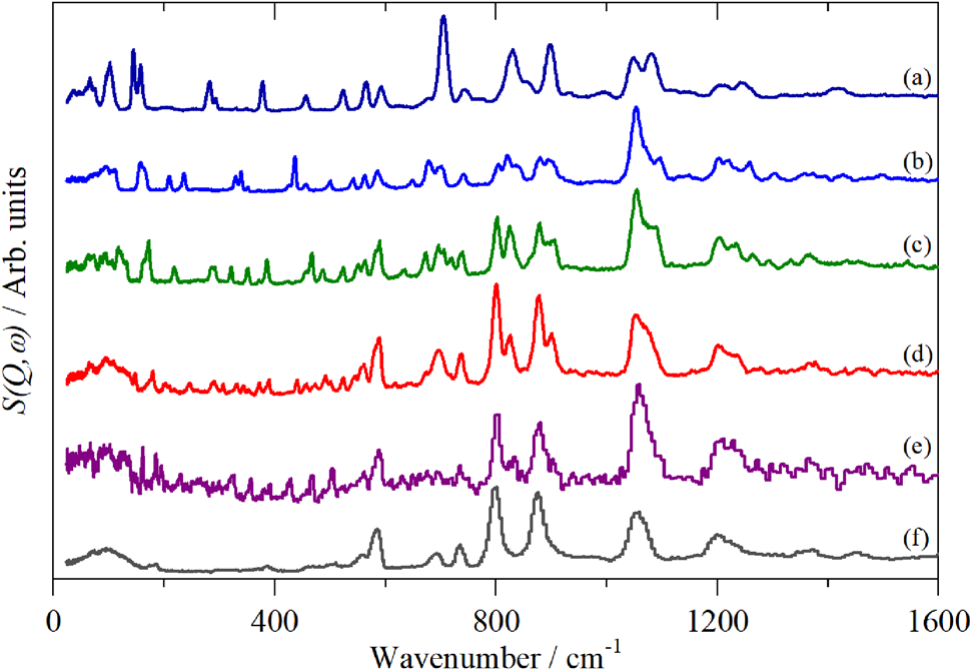
INS spectra of the oligothiophenes and polythiophene. (a) 2T, (b) 3T, (c) 4T, (d) 6T, (e) 8T and (f) PT.

Fig. 2–4 shows the infrared FT-Raman and INS spectra of the materials. The evolution of the spectra of the oligothiophenes to that of polythiophene is readily apparent. As might be expected, the spectra of bithiophene are the most different from that of polythiophene: the former is essentially only the chain termination and the latter is largely composed of linking thiophenes. The spectra of the oligothiophenes have been assigned previously (2T,^10,15,17,26^ 3T,^10^ 4T,^10,14,15,27^ 6T,^14,15,27,55^ 8T ^14^).

The structure of polythiophene contains two chains (four rings) per unit cell.^54,56^ The crystallography shows that the space group is probably *Pna*2_1_ (no. 33) with the “molecules” on a *C*_1_ site. However, the bond distances in all four rings are the same (to three decimal places), thus the structure can be described in the higher symmetry space group *Pnma* (no. 62) with the mid-point of the inter-ring bond on a site of *C*_i_ symmetry. The additional symmetry significantly simplifies the lattice dynamic calculations. The factor group is *D*_2h_ and the modes at the *Γ*-point (*k* = 0) in the Brillouin zone comprise:11*A*_g_ + 10*B*_1g_ + 11*B*_2g_ + 10*B*_3g_ + 10*A*_u_ + 11*B*_1u_ + 10*B*_2u_ + 11*B*_3u_

The presence of two chains in the unit cell means that each mode in the crystal consists of an in-phase and out-of-phase pair, hence the total representation can be simplified to:11(*A*_g_ + *B*_2g_) + 10(*B*_1g_ + *B*_3g_) + 10(*A*_u_ + *B*_2u_) + 11(*B*_1u_ + *B*_3u_)

The symmetry does not permit in-plane and out-of-plane modes to mix, so the representation can be divided into:In-plane = 7(*A*_g_ + *B*_2g_) + 7(*B*_1g_ + *B*_3g_) + 6(*A*_u_ + *B*_2u_) + 6(*B*_1u_ + *B*_3u_)Out-of-plane = 2(*A*_g_ + *B*_2g_) + 3(*B*_1g_ + *B*_3g_) + 2(*A*_u_ + *B*_2u_) + 3(*B*_1u_ + *B*_3u_)

The remaining modes (2(*A*_g_ + *B*_2g_) + 2(*A*_u_ + *B*_2u_) + 2(*B*_1u_ + *B*_3u_)) are translations and librations of the entire chain.

**Fig. 5:**
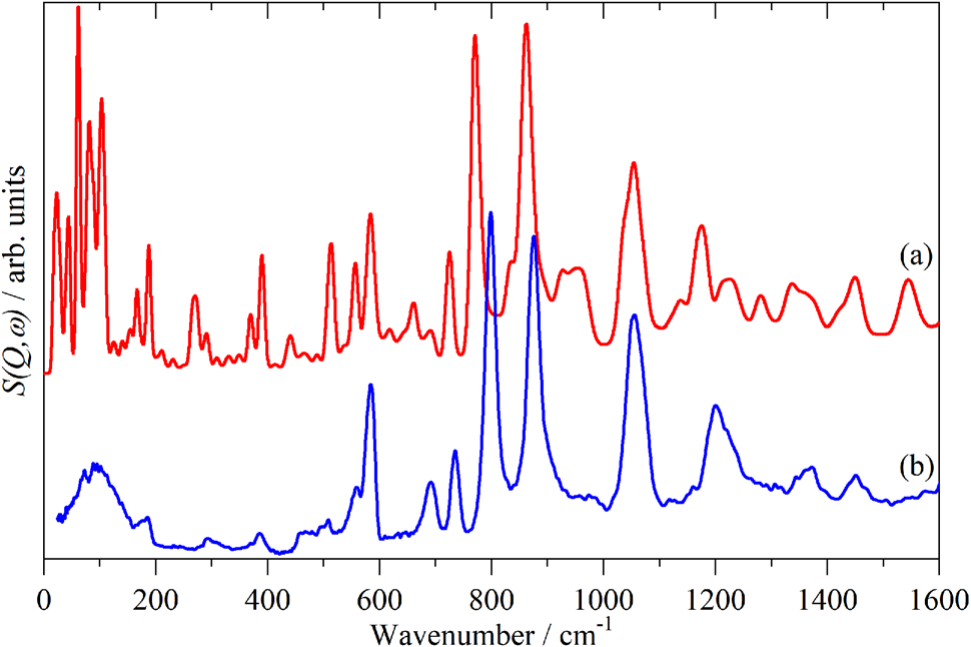
Comparison of the calculated (a) and experimental (b) INS spectra of polythiophene.

**Fig. 6:**
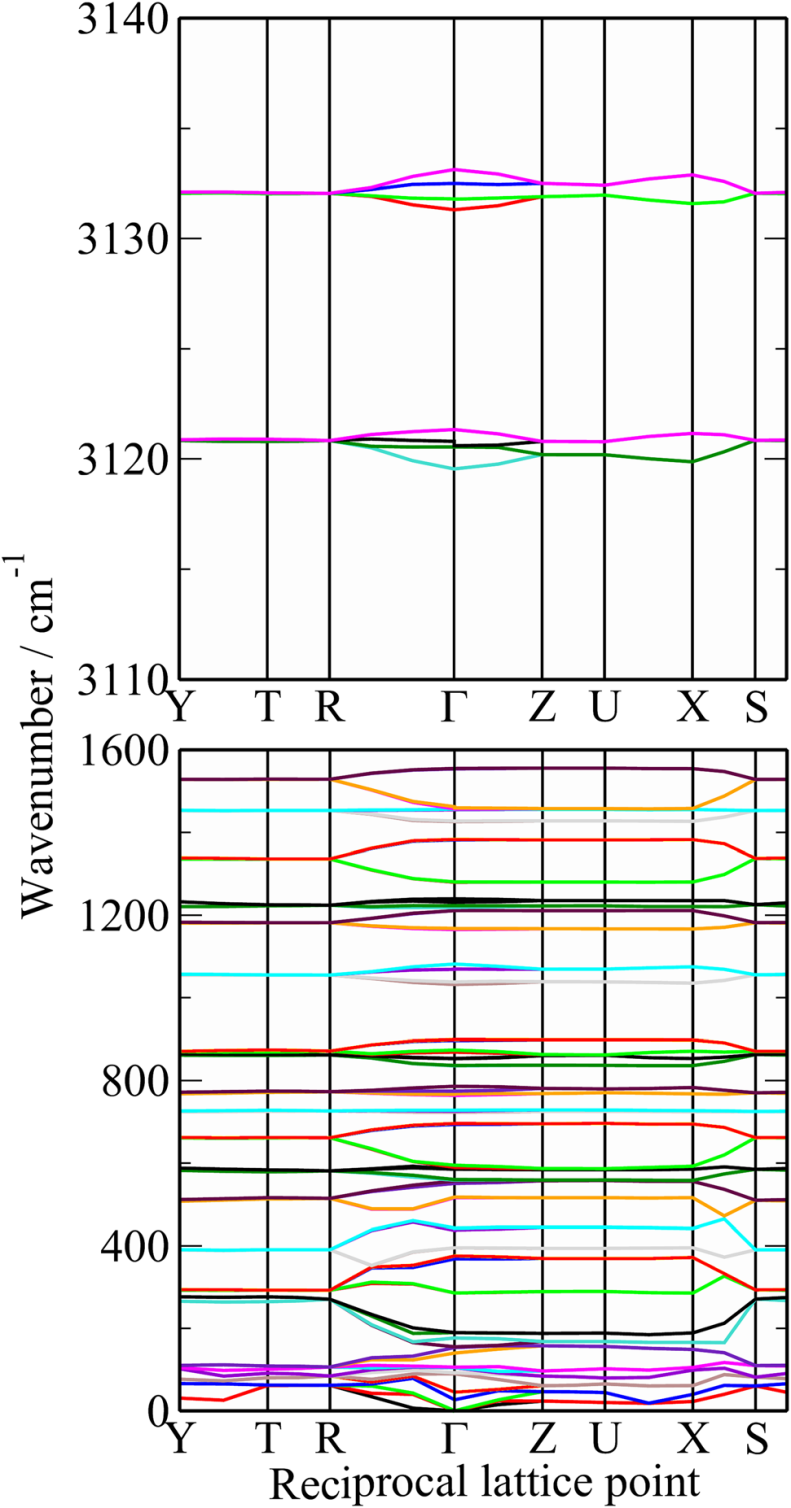
Dispersion curves of polythiophene.

A comparison of a DFT calculation of the INS spectrum for the vibrational modes across the entire Brillouin zone with the experimental INS spectrum is shown in Fig. 5 (INS spectroscopy is sensitive to modes at all wavevectors, *k*, not just those at *k* = 0 as for infrared and Raman spectroscopy^32^). The dispersion curves are shown in Fig. 6. The agreement in Fig. 5 is generally good, particularly in the 500–1600 cm^−1^ region, where both the transition energy and intensity are well-reproduced. In the region below 500 cm^−1^, the transition energies are accurately reproduced but the intensities are somewhat over-estimated. Most of the modes in this region are out-of-plane modes (see Table 1) and if there were some degree of orientation in the sample, this would affect the intensities. (For an INS transition to have any intensity, the momentum transfer vector, 
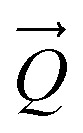
, must be parallel to the direction of motion). In the 0–200 cm^−1^ region the whole-body translations and librations occur. The lack of structure in the experimental spectrum suggests that the sample is poorly crystalline.

**Table 1:** Observed and calculated modes of polythiophene. A complete list of the calculated transition energies with more detailed description is given in Table S1 of the ESI

Observed^a^/cm^−1^			CASTEP^b^/cm^−1^		Description
Infrared	Raman	INS	Average^c^	Range^d^	
58 *w*					Translation
		72 *w*			Translation
100 *w*		100 br, *m*			Translation/libration
		185 *w*	189	0	Inter-ring in-plane deformation
	293 *vw*	293 *w*	286	0	Inter-ring in-plane deformation
		386 *w*	395	0	Inter-ring in-plane deformation
456 *m*		458 *w*	440	5	Intra-ring torsion
		509 *w*	517	1	Inter-ring in-plane deformation
		558 *w*	555	9	Intra-ring torsion
584 *w*		585 *m*	584	0	Inter-ring in-plane deformation
701 *m*	701 *vw*	692 *m*	695	3	Intra-ring in-plane deformation
737 *vw*		735 *m*	727	2	Intra-ring in-plane deformation
786 *s*		799 *s*	780	12	Out-of-plane C–H bend
830 *m*			836	1	Intra-ring in-plane deformation
		876 *s*	871	5	Out-of-plane C–H bend
1044 *w*, 1070 *w*	1047 *vw*	1055 *m*	1075	13	In-plane C–H bend
		1200 *m*	1167	2	In-plane C–H bend
	1368 *vw*	1372 *w*	1382	1	Ring stretch
1425 *vw*, 1439 *w*		1430 *w*	1455	1	Ring stretch
	1456 *vs*	1451 *w*			Ring stretch
3047 sh					C–H stretch
3061 *w*					C–H stretch
3074 *w*					C–H stretch

a *s* = strong, *m* = medium, w = weak, br = broad, sh = shoulder, *v* = very.

b Transition energies at the *Γ*-point of the complete unit cell containing two chains.

c Average of the factor group split transition energies at the *Γ*-point.

d Difference between the highest and lowest transition energy of the factor group at the *Γ*-point.

The dispersion curves in Fig. 6 are largely flat, showing that little dispersion (variation of transition energy with wavevector) is present. This is somewhat surprising as the polymer chain provides an ideal mechanism (connection between neighbouring unit cells) for dispersion to occur, as seen in polyethylene.^57^

Table 1 lists the observed and calculated modes, together with assignments based on the mode visualisation. A more detailed description for each mode is given in Table S1.† It can be seen that the factor group splitting is small for the internal modes: the largest is 13 cm^−1^ and for most of them it is 5 cm^−1^ or less.

### 3.2 The effect of doping on the oligothiophenes and polythiophene

**Fig. 7:**
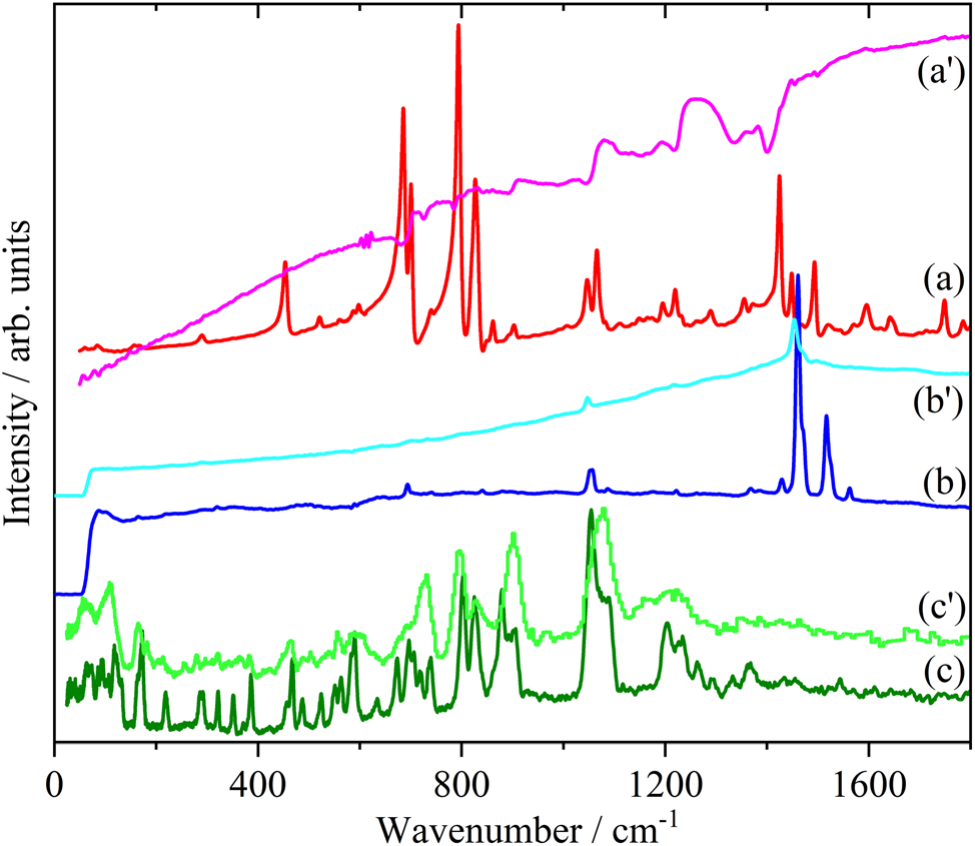
Infrared (a), Raman (b) and INS spectra of 4T. The primes denote the iodine doped material.

**Fig. 8:**
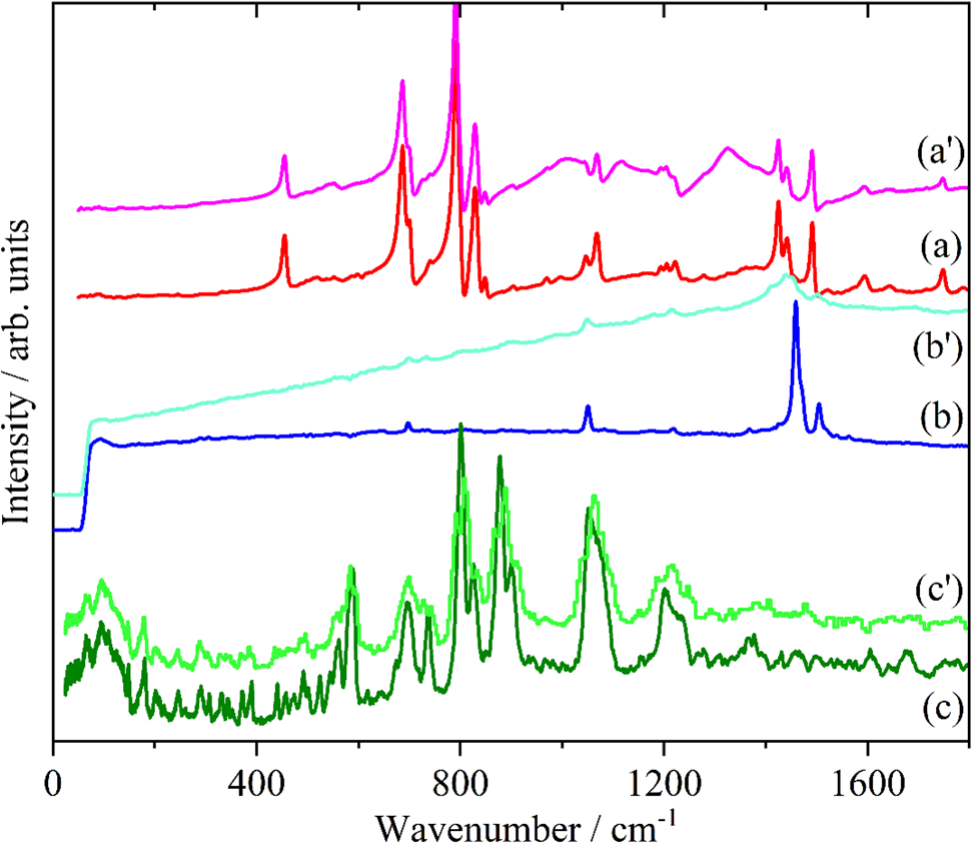
Infrared (a), Raman (b) and INS spectra of 6T. The primes denote the iodine doped material.

**Fig. 9:**
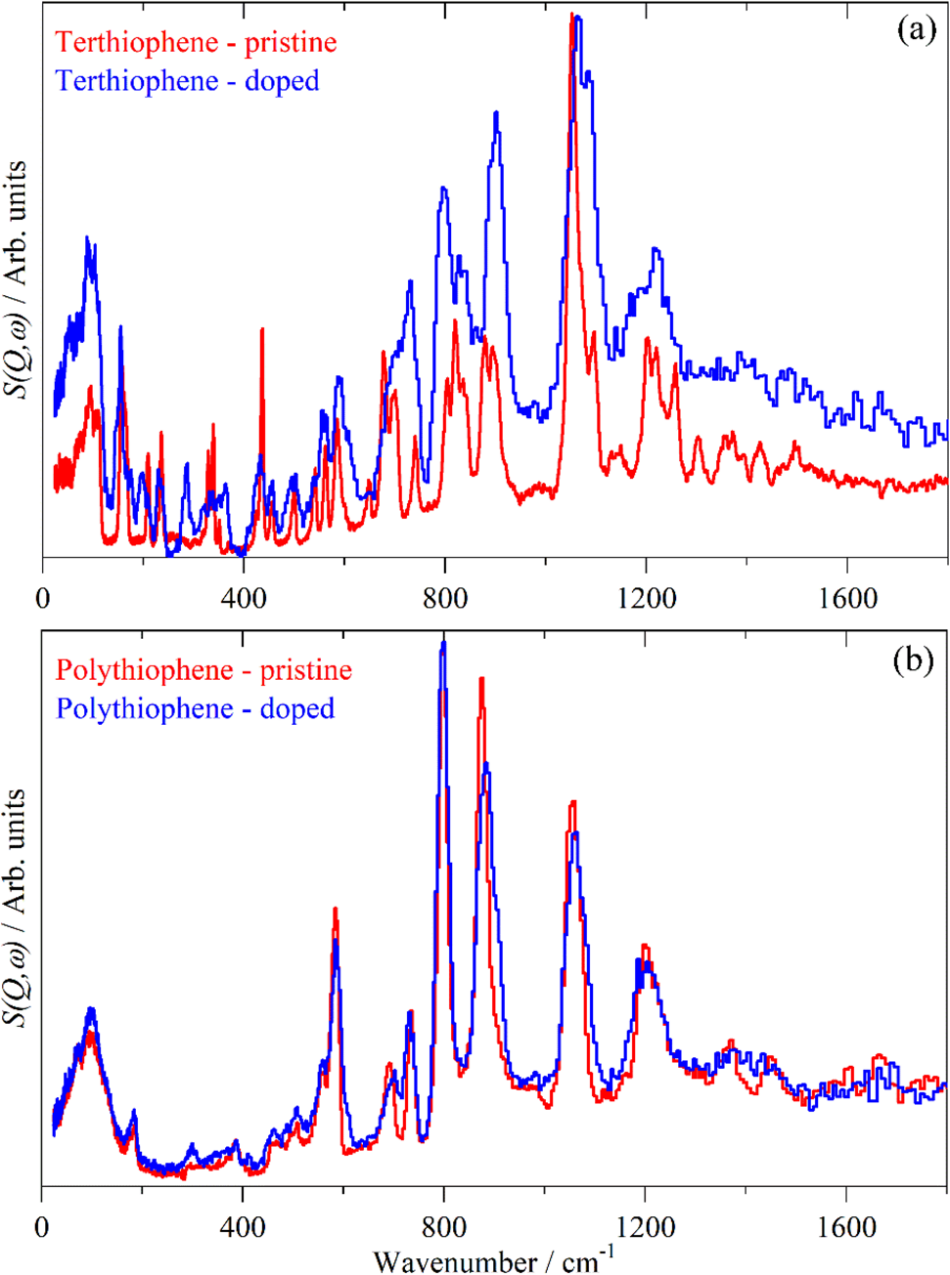
Comparison of the INS spectra of pristine and iodine doped 3T (a) and PT (b).

Doping of the oligothiophenes or polythiophene with either electron donors or acceptors produces large increases in conductivity.^8^ The changes in the electronic structure caused by doping result in dramatic changes in the infrared and Raman spectra as shown for pristine and I_2_ doped 4T and 6T in Fig. 7 and 8 respectively. These represent a heavily doped (4T: 1.38 I per T ring) and a lightly doped (6T: 0.20 I per T ring) system. In contrast, the INS spectra of the same systems (Fig. 7c, c’ and 8c, c’) and those of 3T and PT (Fig. 9) show relatively minor changes in the spectra. 4T shows the largest changes but these are more like a loss of resolution than new bands appearing. The INS spectrum of doped PT only shows changes in relative intensity of the modes. The band shifts are only a few cm^−1^ at most.

There are a number of possible reasons why the INS spectra are relatively insensitive to doping. It may be that doping results in a core–shell particle structure with a thin layer of doped material while the bulk is untouched. As neutrons are highly penetrating, the spectra will be an average of the shell and the core, with the latter dominating owing to its greater proportion. We do not consider this likely as PT is poorly crystalline: values of 35 ^56^ to 66%^58^ crystallinity are quoted and halogens readily permeate amorphous regions of polymers, as seen for polyethylene^59^ and polyisoprene.^60^

The most likely reason is that the INS intensities do not depend on the electronic structure in the same way that infrared and Raman spectroscopy do. The mode intensity for the latter two depends on changes in electronic properties (dipole moment and polarizability respectively) whereas for INS it is determined by the amplitude of motion of the atoms in the mode and their cross section. The former is a function of the molecular structure, if this is largely unchanged on doping, then the INS spectrum will also be largely unchanged. This is exemplified in the series C_60_, K_3_C_60_ and Rb_6_C_60_ which contain C_60_, [C_60_]^3−^ and [C_60_]^6−^ entities that all exhibit very similar INS spectra.^61^

From Fig. 2–4, it is apparent that the spectra of the oligothiophenes quickly evolve towards that of polythiophene, such that 6T and 8T strongly resemble that of PT. To investigate the effect of doping on the INS spectrum of PT, we have used 4T and 6T as model compounds to make the calculations as function of doping level feasible in a reasonable time. These were also chosen because our experimental data represent a heavily doped and a lightly doped (4T: 1.38, 6T: 0.20 iodine per thiophene ring) system. We have calculated 4T with charges from 0 to +4 (denoted 4T0, 4T + 1, 4T + 2 *etc*…) and 6T from 0 to +6 (denoted 6T0, 6T + 1, 6T + 2 *etc*…).

**Fig. 10:**
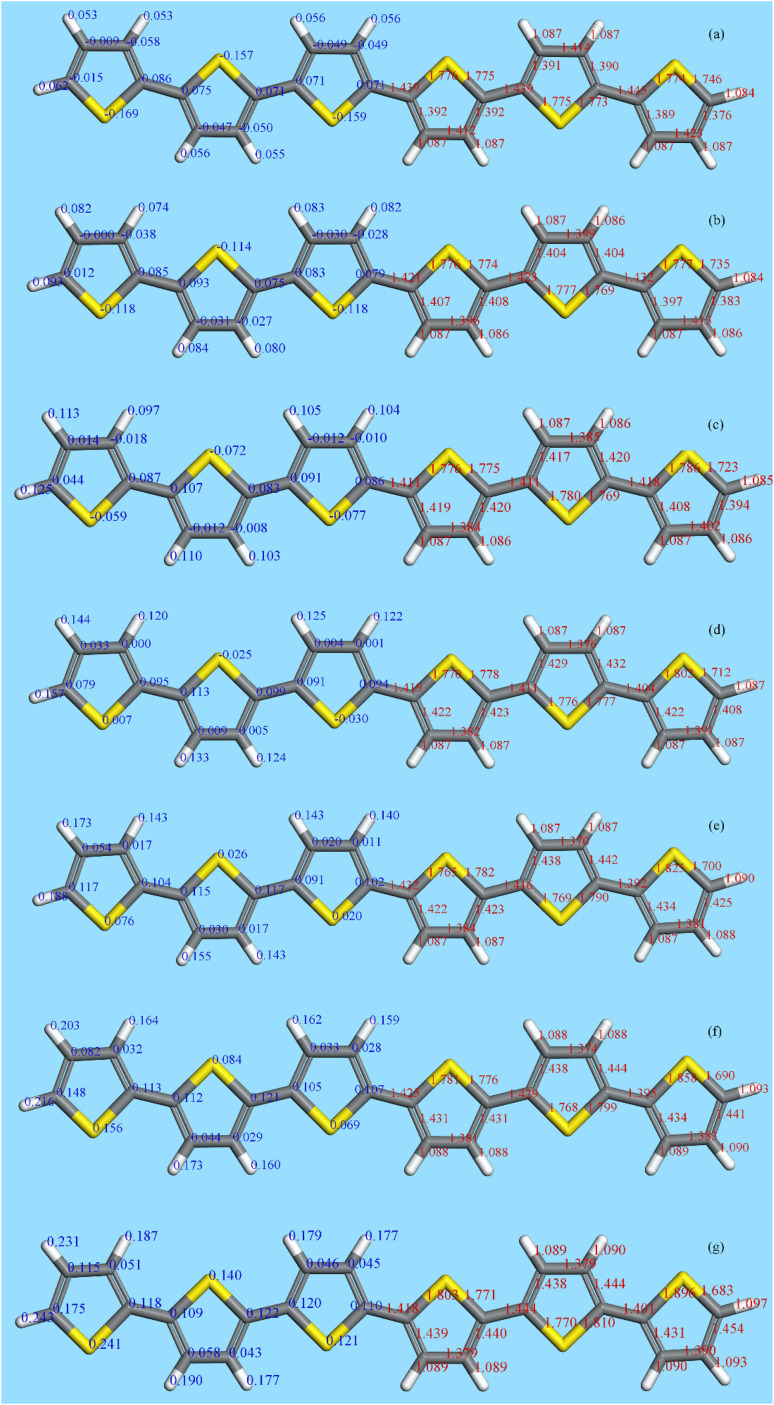
The structure (right hand side, red numbers (Å)) and Mulliken charges (left hand side, blue numbers (e)) of 6T (note that it is centrosymmetric). (a) Neutral molecule, (b) +1, (c) +2, (d) +3, (e) +4, (f) +5 and (g) +6.

**Fig. 11:**
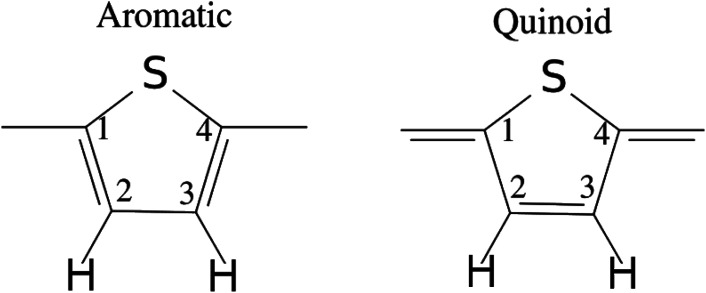
Illustration of the benzenoid and quinoid structures in PT. Adapted from ref. 66 under a Creative Commons Attribution 4.0 International License.

Fig. 10 shows the results for 6T (4T is similar). The left side shows the Mulliken charges (in blue) and the right the bond distances (in red). Note that 6T is centrosymmetric. Concerning the charges, in the neutral molecule, 6T0, the carbon and hydrogen atoms are slightly positive and the sulfur atoms are slightly negative. As the charge increases, all the atoms become more positive with the endmost atoms being the most positive. However, the charge is distributed across all of the atoms, it is not localised, although the endmost atoms experience the largest increase. This is mirrored in the bond lengths; all of the C–C and C–S distances increase on doping, except for the C2–C3 bond (see Fig. 11) which shrinks, although the change is small (∼+0.01 Å) for the C–H bondlengths.

There have been many calculations of the effect of doping on the structure of the oligothiophenes, PT and related materials *e.g*.^62–66^ There is general agreement that doping induces the quinoid-type structure from the aromatic structure of the neutral systems (see Fig. 11 for an illustration of the structures) and our results are consistent with this picture. However, most of the discussion has been in terms of localised defects and we do not see this, rather the charge is distributed across the entire molecule.

**Fig. 12:**
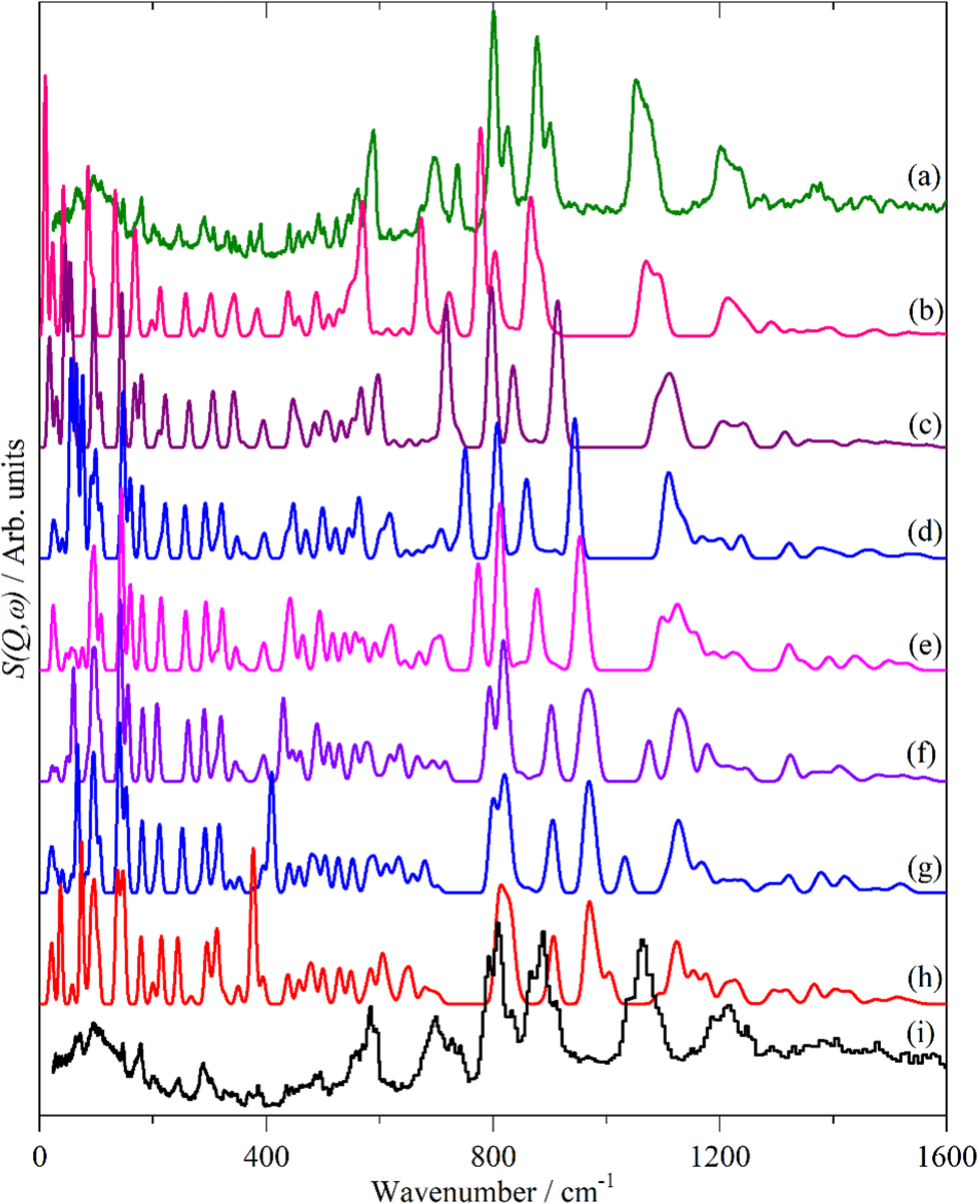
Observed and calculated INS spectra of doped 6T. (a) Experimental neutral molecule spectrum, calculated spectra of (b) neutral, (c) +1, (d) +2, (e) +3, (f) +4, (g) +5, (h) +6 and (i) experimental I_2_-doped molecule spectrum.

**Fig. 13:**
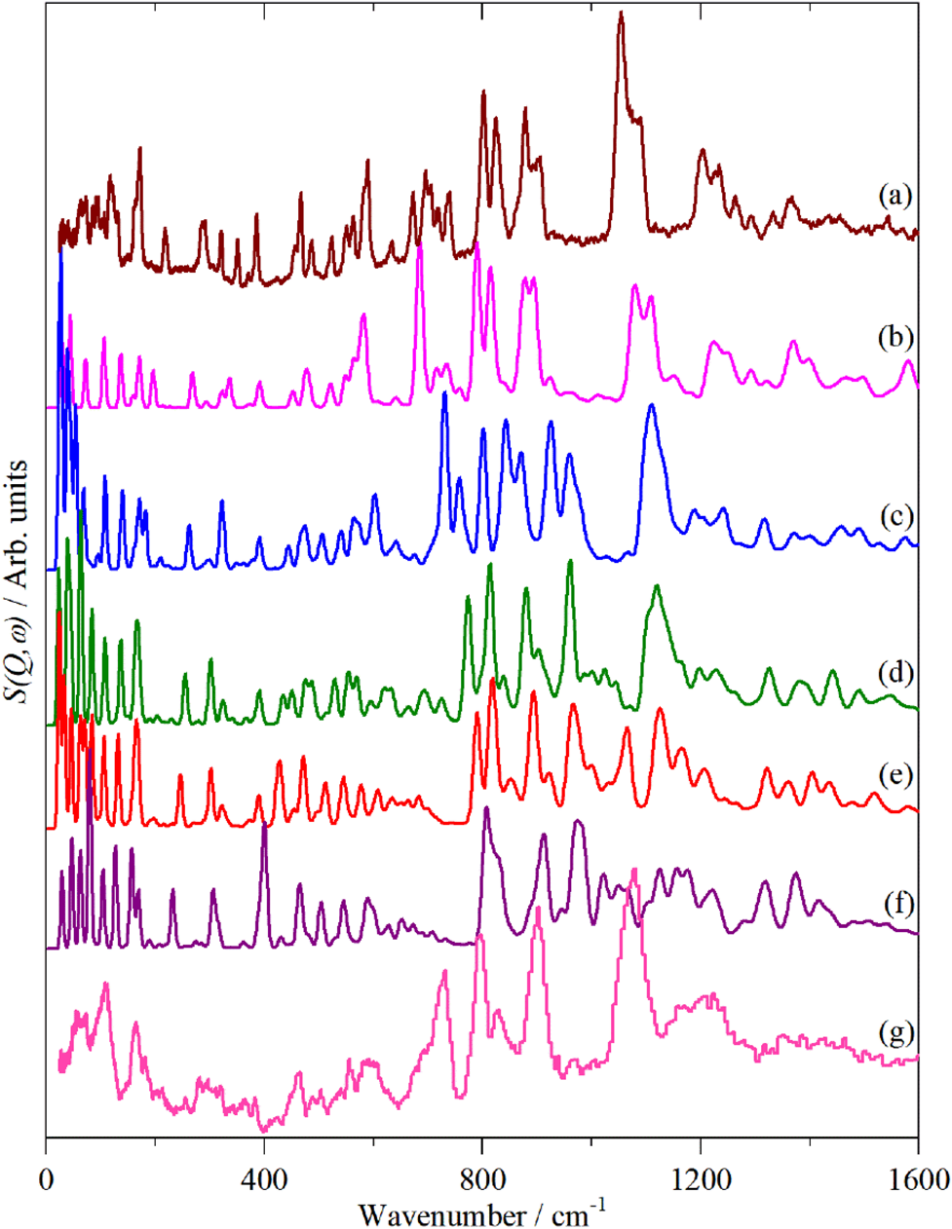
Observed and calculated INS spectra of doped 4T. (a) Experimental neutral molecule spectrum, calculated spectra of (b) neutral, (c) +1, (d) +2, (e) +3, (f) +4 and (g) experimental I_2_-doped molecule spectrum.

Fig. 12 and 13 compare the experimental INS spectra (neutral and doped) with those calculated for 6T and its ions and 4T and its ions, respectively. The first point to note is that the experimental and calculated spectra of the neutral ions (Fig. 12a, b and 13a, b) are in good agreement, which provides confidence that the calculations are reliable. In both systems, it can be seen that the modes around 700 cm^−1^ shift to higher energy and merge into those around 800 cm^−1^. Visualisation of the modes shows that those near 700 cm^−1^ are in-plane ring deformations and those around 800 cm^−1^ are out-of-plane C–H bending modes. The increase in the in-plane modes suggests that the quinoid structure is stiffer than the parent aromatic structure. The constancy of the C–H bending modes (and also of the C–H stretch) modes is consistent with the very small changes in C–H bond length that occur on doping.

6T represents a lightly doped system (0.2 iodine-per-thiophene ring) and inspection of Fig. 12 shows that the best match for the doped spectrum (Fig. 12i) is intermediate between the neutral system (6T0, Fig. 12b) and the +1 system (6T + 1, Fig. 12c). 4T is a much more highly doped system (1.38 iodine-per-thiophene ring) and the change in the INS spectrum is more marked (*c.f.*Fig. 13a and g). By inspection, the best match is intermediate between the +1 and +2 systems (Fig. 13c and d). This represents 0.25–0.5 iodine-per-thiophene ring, which indicates that not all of the dopant has been effective. We examined the Raman spectra of the doped materials for evidence of polyiodide anions or unreacted I_2_ but did not find any.

## 4. Conclusions

In this paper we have measured the vibrational spectra (infrared, Raman and INS) of a series of oligothiophenes and polythiophene, both pristine and after doping with iodine. The pristine (*i.e*. neutral) systems show a rapid convergence towards the spectrum of PT, such that the spectra of 6T and 8T are almost indistinguishable from that of polythiophene.

In contrast to the infrared and Raman spectra that show dramatic changes on doping, the INS spectra show only small changes. Isolated molecule DFT calculations show that the molecular structures are not greatly modified on doping and since the INS spectrum largely depends on the structure, this does not change much. In contrast, as shown by others,^62–66^ the electronic structure is greatly modified and this accounts for the major changes in the infrared and Raman spectra.

## Conflicts of interest

There are no conflicts of interest to declare.

## Supplementary Material

RA-013-D2RA07625J-s001
