# Supplementary material for: Vibrational spectra of neutral and doped oligothiophenes and polythiophene
Source: RSC Adv. 2023 Feb 13;13(8):5419–27. doi: 10.1039/d2ra07625j (PMC9923818; doi:10.1039/d2ra07625j)
Supplement: RA-013-D2RA07625J-s001 [file RA-013-D2RA07625J-s001.pdf]

Electronic Supplementary Material (ESI) for RSC Advances.
This journal is © The Royal Society of Chemistry 2023

Electronic supplementary information for:

Vibrational spectra of neutral and doped oligothiophenes and polythiophene

Stewart F. Parker,* Jessica E. Trevelyan and Hamish Cavaye

ISIS Neutron and Muon Source, STFC Rutherford Appleton Laboratory, Chilton, Didcot, OX11 0QX, UK
*Author to whom correspondence should be addressed. E-mail: stewart.parker@stfc.ac.uk

Characterisation of polythiophene

The polythiophene (PT) used in this work was purchased from Reike Metals. PT is insoluble in all common solvents, so it is not possible to characterise the molecular weight by conventional methods, such as gel permeation chromatography (GPC). However, there are some empirical correlations between the degree of polymerisation, conjugation length and the infrared and Raman spectra [1-3].

In the infrared spectrum, the ratio of the bands at 690 and 790 cm^{-1} (0.32, assigned to chain ends and internal rings respectively) corresponds to a degree of polymerisation (N_{dp}) of 8.7 [1,2]. This is certainly an underestimate as it would predict that the spectra of 8T and PT would be almost identical. Inspection of Figures 2-4, shows that this is not the case, so the sequence length must be longer.

Similarly, the ratio of the C1=C2 and C3=C4 antisymmetric (out-of-phase, 1490 cm^{-1}) and symmetric (in-phase, ~1439 cm^{-1}) stretching modes can be used to determine the conjugation length (N_c) [1,2] (See Figure 11 for the numbering). For this material, the ratio is 0.77, corresponding to a conjugation length >6. The line width of the symmetric mode in the Raman spectrum is inversely correlated with N_c [3]. The observed line width (16 cm^{-1}) is consistent with the infrared results and implies a high degree of conjugation.

In the Raman spectrum, bands at 652, 682, 1155 and 1177 cm^{-1} "arise from the distorted parts in the polymers" [3]. None of these are seen in our sample.

Together, these results indicate that our sample shows a relatively low degree of polymerisation but the resulting material is highly conjugated (i.e. it exhibits the alternating S-up, S-down, S-up conformation shown in Figure 1) and is essentially defect free. We note that the conductivity of the doped materials is more correlated with N_c than N_{dp} [1].

[1] M. Akimoto, Y. Furukawa, H. Takeuchi, I. Harada, Y. Soma and M. Soma, *Synth. Metals* 1986, **15**, 353-360

[2] Y. Furukawa, M. Akimoto and I. Harada, *Synth. Metals*, 1987, **18**, 151-156.

[3] J. L. Sauvajol, G. Poussigue, C. Benoit, J. P. Lère-Porte and C. Chorro, *Synth. Metals*, 1997, **84**, 569-570.

Table S1 Assignment of the vibrational spectrum of polythiophene from a periodic-DFT calculation of the complete unit cell (space group *P*nma, *Z* = 2) at the Γ-point of the Brillouin zone.

Mode number	Transition energy / cm^{-1}	Symmetry	IR activity / km mol^{-1}	IR allowed?	Raman allowed?	Average[a] / cm^{-1}	Range[b] / cm^{-1}	Description
1	0	B1u	0.00	N	N	0	0	Acoustic mode
2	0	B2u	0.00	N	N	13	27	Acoustic mode
3	0	B3u	0.00	N	N			Acoustic mode
4	27	Au	0.00	N	N			Translation
5	45	B3u	0.06	Y	N	69	47	Translation
6	90	Ag	0.00	N	Y			Libration
7	92	B1u	1.65	Y	N	97	15	Translation
8	105	B1u	0.02	Y	N	105	1	Inter-ring torsion
9	105	B2g	0.00	N	Y			Libration
10	106	B3u	6.17	Y	N			Inter-ring torsion
11	140	Ag	0.00	N	Y	148	15	Libration
12	154	B2u	1.84	Y	N	165	23	Libration
13	155	B2g	0.00	N	Y			Libration
14	177	Au	0.00	N	N			Libration
15	189	Au	0.00	N	N	189	0	Inter-ring in-plane deformation
16	190	B2u	9.85	Y	N			Inter-ring in-plane deformation

17	286	Ag	0.00	N	Y	286	0	Inter-ring in-plane deformation
18	286	B2g	0.00	N	Y			Inter-ring in-plane deformation
19	368	B1g	0.00	N	Y	372	7	Intra-ring torsion
20	376	B3g	0.00	N	Y			Intra-ring torsion
21	395	B3g	0.00	N	Y	395	0	Inter-ring in-plane deformation
22	395	B1g	0.00	N	Y			Inter-ring in-plane deformation
23	437	B1u	24.81	Y	N	440	5	Intra-ring torsion
24	442	B3u	60.44	Y	N			Intra-ring torsion
25	516	B3g	0.00	N	Y	517	1	Inter-ring in-plane deformation
26	518	B1g	0.00	N	Y			Inter-ring in-plane deformation
27	551	Au	0.00	N	N	555	9	Intra-ring torsion
28	556	B2g	0.00	N	Y			Intra-ring torsion
29	559	Ag	0.00	N	Y	557	2	Intra-ring torsion
30	560	B2u	3.25	Y	N			Intra-ring torsion
31	585	B1u	0.31	Y	N	585	0	Inter-ring in-plane deformation
32	585	B3u	0.01	Y	N			Inter-ring in-plane deformation
33	590	B3g	0.00	N	Y	592	5	Intra-ring torsion

34	595	B1g	0.00	N	Y			Intra-ring torsion
35	693	Ag	0.00	N	Y	695	3	Intra-ring in-plane deformation
36	696	B2g	0.00	N	Y			Intra-ring in-plane deformation
37	723	B1g	0.00	N	Y	724	2	Intra-ring in-plane deformation
38	725	B3g	0.00	N	Y			Intra-ring in-plane deformation
39	726	B2u	33.02	Y	N	727	2	Intra-ring in-plane deformation
40	728	Au	0.00	N	N			Intra-ring in-plane deformation
41	764	B3u	365.70	Y	N	764	0	Out-of-plane C-H bend
42	764	B1u	130.22	Y	N			Out-of-plane C-H bend
43	774	Ag	0.00	N	Y	780	12	Out-of-plane C-H bend
44	786	B2g	0.00	N	Y			Out-of-plane C-H bend
45	835	B1u	42.63	Y	N	836	1	Intra-ring in-plane deformation
46	836	B3u	25.55	Y	N			Intra-ring in-plane deformation
47	853	B2u	0.25	Y	N	853	1	Out-of-plane C-H bend
48	854	Au	0.00	N	N			Out-of-plane C-H bend
49	869	B1g	0.00	N	Y	871	5	Out-of-plane C-H bend
50	874	B3g	0.00	N	Y			Out-of-plane C-H bend

51	897	B2u	46.97	Y	N	898	2	In-plane C-H bend
52	899	Au	0.00	N	N			In-plane C-H bend
53	1032	Ag	0.00	N	Y	1035	6	In-plane C-H bend
54	1038	B2g	0.00	N	Y			In-plane C-H bend
55	1069	B1u	52.25	Y	N	1075	13	In-plane C-H bend
56	1081	B3u	28.09	Y	N			In-plane C-H bend
57	1165	Au	0.00	N	N	1167	2	In-plane C-H bend
58	1168	B2u	64.20	Y	N			In-plane C-H bend
59	1211	B3g	0.00	N	Y	1211	0	Intra-ring in-plane deformation
60	1211	B1g	0.00	N	Y			Intra-ring in-plane deformation
61	1219	B2g	0.00	N	Y	1221	4	Intra-ring in-plane deformation
62	1223	Ag	0.00	N	Y			Intra-ring in-plane deformation
63	1231	B1u	10.49	Y	N	1235	8	Intra-ring in-plane deformation
64	1240	B3u	0.46	Y	N			Intra-ring in-plane deformation
65	1280	B1g	0.00	N	Y	1280	1	Inter-ring stretch
66	1281	B3g	0.00	N	Y			Inter-ring stretch
67	1382	B2g	0.00	N	Y	1382	1	Ring stretch
68	1383	Ag	0.00	N	Y			Ring stretch
69	1426	B2g	0.00	N	Y	1427	2	Ring stretch

70	1428	Ag	0.00	N	Y			Ring stretch
71	1455	B1u	30.97	Y	N	1455	1	Ring stretch
72	1456	B3u	17.89	Y	N			Ring stretch
73	1457	Au	0.00	N	N	1458	2	Ring stretch
74	1459	B2u	1375.97	Y	N			Ring stretch
75	1554	B1g	0.00	N	Y	1554	1	Ring stretch
76	1555	B3g	0.00	N	Y			Ring stretch
77	3120	Au	0.00	N	N	3120	1	Out-of-phase C-H stretch
78	3121	B1g	0.00	N	Y	3121	1	Out-of-phase C-H stretch
79	3121	B2u	196.83	Y	N			Out-of-phase C-H stretch
80	3121	B3g	0.00	N	Y			Out-of-phase C-H stretch
81	3131	B1u	0.35	Y	N	3132	0	In-phase C-H stretch
82	3132	B3u	19.26	Y	N			In-phase C-H stretch
83	3132	Ag	0.00	N	Y	3133	1	In-phase C-H stretch
84	3133	B2g	0.00	N	Y			In-phase C-H stretch

[a] Average of the factor group split transition energies at the Γ-point.

[b] Difference between the highest and lowest transition energy of the factor group at the Γ-point.
